# Homeownership, Depression, and Life Satisfaction in China: The Gender and Urban-Rural Disparities

**DOI:** 10.3390/ijerph192214833

**Published:** 2022-11-11

**Authors:** Bo Kyong Seo, In Hyee Hwang, Yi Sun, Juan Chen

**Affiliations:** 1 Department of Applied Social Sciences, Centre for Social Policy and Social Entrepreneurship, The Hong Kong Polytechnic University, Hong Kong, China; 2 Department of Political Science, Sogang University, Seoul 04107, Korea; 3 Department of Building and Real Estate, Research Institute for Land and Space, The Hong Kong Polytechnic University, Hong Kong, China; 4 Department of Applied Social Sciences, Mental Health Research Centre, The Hong Kong Polytechnic University, Hong Kong, China

**Keywords:** homeownership, depressive symptoms, life satisfaction, China, regional gap, gender difference

## Abstract

This study examines how depression and life satisfaction are associated with assets in the form of homeownership in China and whether their relationships differ between men and women, and between urban and rural areas. While the psychological benefits of homeownership are well-documented, how gender makes a difference in this relationship remains unclear. Given the dynamic housing market conditions characterized by the urban-rural divide and the notable gender gap in psychological well-being, China can provide a relevant context to address this knowledge gap. A series of linear regression analyses based on the China Family Panel Studies (CFPS) data show that homeownership is positively associated with life satisfaction and negatively related to depression, and this relationship is driven by men. While the homeownership-life satisfaction relation does not differ between urban and rural areas, the negative association between homeownership and depression is seen only among rural residents. The gender difference could be explained by the salient role of the financial security obtained from homeownership, whereas the regional difference seems to be supported by the social comparison theory. This study contributes to the knowledge of how a biological determinant, i.e., gender, interacts with a social determinant, i.e., homeownership, to affect psychological well-being.

## 1. Introduction

Depression and life satisfaction are two key dimensions of psychological well-being. Depression has an adverse effect on people’s daily functioning, often leading to suicide [1], and life satisfaction is an indicator of subjective well-being and good health [2]. However, about 280 million people are suffering from depression worldwide, and mental health problems have constantly escalated [3]. Therefore, how to prevent mental health risks has received increasing policy attention.

There are a multitude of studies on the determinants of depression and life satisfaction (for example, [4,5,6,7,8,9,10,11]). The literature suggests that psychological well-being is influenced by biological (e.g., genetic difference, hormones), psychological (e.g., personality, job stress, resilience), social (e.g., socio-economic status, social support), and behavioural (e.g., help-seeking behaviours, mood amplification) factors [1,12,13,14,15]. With regard to the social factors, it has been consistently found that low socio-economic status (SES) is associated with poor health [1,16,17]. Among the various SES conditions, exposure to financial difficulties that resulted from low income has been consistently found to be the major SES risk of psychological well-being (e.g., [9,17,18,19,20]). However, it has been pointed out that income may not reflect one’s financial resources accurately, since it only captures the flow of money when it is measured [21]. There has been growing interest in ‘*assets*’ as another key indicator of one’s material wealth that influences psychological well-being [21,22]. Assets indicate the material richness that has been accumulated over time. Recognising the subtle difference between income and assets as financial resources, Sherraden [21] aptly stated that ‘while incomes feed people’s stomachs, assets change their heads (p. 6)’. This seems to imply that asset ownership conveys not only financial benefits in the economic aspect, but also psychological effects conducive to psychological well-being [23].

This study attempts to discern this assumption by examining the role of homeownership, the largest share of a household’s assets [24], in influencing psychological well-being. In fact, extensive research has already found a significantly positive association between homeownership and psychological well-being [25,26,27,28]. However, relatively little is known about whether gendered differences are at play in this relationship. Given that men and women tend to show different patterns in some of the mental health dimensions [9,12,29,30], as well as in the attitudes toward financial resources [31,32], it can be postulated that the influence of homeownership on psychological well-being differs across genders. Yet gender has tended to be merely used as a control variable and has been rarely included as a primary predictor in prior research on the health effects of homeownership (or assets more broadly) [33,34]. This knowledge gap is likely to hinder the development of effective interventions to promote psychological well-being.

Therefore, we aim to provide empirical evidence to identify how depression and life satisfaction are associated with homeownership and whether their relationships vary by gender, using the nationally representative household survey data in China. Since the housing reform in the 1990s, housing assets have contributed significantly to wealth accumulation among homeowners in China [35]. However, there has been a clear disparity between urban and rural housing markets which has brought about considerable wealth inequality [36]. Therefore, we investigate whether the relationship among homeownership, psychological well-being, and gender differs between urban and rural areas.

In the following sections, we first give an overview of the theoretical discussions on the relationship between homeownership, gender, and psychological well-being to formulate the hypotheses to be investigated. After introducing the data and analytic strategies we used to examine the hypotheses, we report the analysis results and discuss the key findings in relation to the literature and empirical context. Finally, we conclude with the relevant implications and limitations of this study.

## 2. Literature Review

### 2.1. Gender Differences in Psychological Well-Being: How Are They Related to Assets in the Form of Homeownership?

Depression is one of the most widely studied dimensions of psychological well-being in gender disparity and is known to be more prevalent among women than men [12]. Life satisfaction, another important dimension of psychological well-being, is negatively associated with depression [37] but shows inconsistent results when it comes to gender differences [30]. While epidemiological studies have focused on biological or behavioural factors in shaping the gender differences in mental health, Denton and Walters [38] claim that structural social gradients are more strongly associated with the gender gap in health outcomes than behaviours or lifestyles are. In particular, many studies drawing on the social causation theory have demonstrated that low SES characterised by low education level, unemployment, and low income is closely related to poor mental health [19,39,40,41,42].

However, what is less well-known is how SES interplays with gender in shaping psychological well-being. While it is agreed that men and women are exposed to different psychological risks and have different psychological resources to handle them [43], whether the association between SES and health differs between men and women is inconclusive due to the lack of empirical evidence and inconsistent patternings in different studies [44] (see, for example, [38,45]). Macintyre and Hunt [43] argued that the association between SES and health is steeper among men than women, meaning that the impact of SES on health is more significant in men than in women. In contrast, Denton et al. [34] and Chun et al. [46] claim that SES is more influential to women’s health since men’s health is more subject to health-related behaviours (e.g., alcohol consumption, smoking). Meanwhile, Back and Lee [47] suggest that while men’s depression is associated with wealth, women’s is linked to education and income. These diverging findings could be explained in part by the types of SES and cultural differences. Yet we need more empirical evidence to establish this assumption.

The asset effect theory [5,48,49,50] suggests that assets bring substantial psychological benefits to the asset holders, and people with no or limited assets are more likely to bear mental distress than those with more assets. Asset theory acknowledges that wealth, one of the important SES indicators, has a substantial impact on psychological outcomes regardless of household income. In particular, owner-occupied housing is an important component of household assets that convey crucial psychological meanings to the residents [51,52,53,54]. The positive effects of homeownership on psychological well-being have been well documented. Scholars have agreed that homeowners tend to have better mental health compared to non-homeowners [25,26,27] unless they experience mortgage burden or foreclosure [8,33,52,55] and locational disadvantages [56]. Prior studies have shown that homeownership offers, or indicates, material resources conducive to maintaining homeowners’ good health. Home purchasing requires sufficient savings to cover a down payment and a steady flow of income above a certain level is required to repay mortgage loans [24]. Outright homeowners expend much less on housing than renters do and thus tend to save more after-housing income that can be used to promote health. It has also been found that renters suffering from housing insecurity are more likely to experience food insecurity [57] and have poor access to health care [58], which are likely to result in poor health. The material advantage of homeownership is believed to help people secure an adequate housing environment that has fewer health risks [59]. Furthermore, homeownership gives owners more favourable psychological resources, such as life satisfaction, happiness, and a sense of belonging [27,56]. Hiscock et al. [60] proved that homeowners, relative to renters, have more ontological security and enjoy psychosocial benefits from their owner-occupied housing. One study showed that older homeowners have a lower likelihood of having depressive symptoms than the elderly who do not own a home, even though the social systems to monetize their property assets for individual welfare are immature [50].

However, Ronald [61] argues that homeownership gives social and psychological benefits to men and women differently. He suggests that while men appreciate the financial security derived from homeownership, women value a sense of stability achieved from owner-occupation. This view can be related to Macintyre’s [62] classification of the ‘materialist’ explanation for health inequality—that is, the material conditions of life could affect health either in a ‘hard’ version (e.g., income, wealth) or in a ‘soft’ version (e.g., psychosocial and physical factors related to the material conditions). Taking Ronald’s [61] and Macintyre’s [62] views together, we could assume that the association between homeownership and psychological well-being may differ between men and women. However, this hypothesis has not been sufficiently examined so far. Park et al.’s [33] study was an exception and found that a higher level of life satisfaction and a lower level of depression among homeowners, relative to renters, were driven by men, not by women. Yet we need more empirical evidence to further validate this finding in other contexts.

### 2.2. Homeownership and Psychological Well-Being in China

China’s market-oriented housing reform since 1998 has significantly increased the homeownership rate. More than 90% of households are currently homeowners in China [63]. Amid the decreasing housing affordability in recent years, higher income and education levels have increasingly become critical SES aspects contributing to homeownership [64]. Prior research has shown that owning a home with no or low financial risk is an overarching protective factor of psychological well-being in China. Particularly in urban China, homeownership is found to be correlated with a lower level of depression [49] and a higher level of subjective well-being [56,65] regardless of the financial constraints induced by home purchase [55]. Hu [56] found that the positive relation between homeownership and subjective well-being is stronger in large cities than in small cities, possibly due to the higher housing prices in large cities. Liang [66] demonstrated that homeownership is not associated with older persons’ depression trajectory in rural China because rural homeownership is less related to wealth accumulation and is based on the relatively equitable residential land allocation under the village’s collective land management system.

The salient linkage between homeownership and psychological well-being in urban China seems attributable to the urban-rural divide in the housing markets. Homeownership has contributed significantly to housing wealth accumulation in home-owning households in China [35]. The commodification of housing and acknowledgement of private property since the housing reform has been driven mainly by urban areas amid the rapid housing price appreciation, and the local Household Registration (*hukou*) system had largely barred migrants from home purchases in urban areas [63]. Consequently, housing wealth inequality has been intensified between urban homeowners and rural homeowners in China [36].

However, rural settlements have continued to expand in the 1980s and 90s along with rural economic growth, and the increased financial capacity has enabled rural-to-urban migrants to reinvest in rural housing construction as part of their plans to return to their homes in the future [67]. As a result, the urban-rural divide in the housing markets has been slowly decreasing alongside the rural land policy reform since the early 2000s. Furthermore, while housing wealth inequality between urban and rural areas persists, housing wealth inequality in rural areas is greater than that in urban areas due to the heterogeneous informal housing markets across different villages [36]. This significant inequality in housing wealth in rural areas may have an adverse influence on psychological well-being among rural residents, in contrast to Liang’s [66] study focusing on older persons who might be less exposed or sensitive to such inequality.

Our study addresses two research questions: (1) Does the association between homeownership and psychological well-being (i.e., depression and life satisfaction) differ between men and women in China?; and (2) Does this association also differ between owners of urban housing and those of rural housing? As non-local *hukou* holders who meet certain criteria are now allowed to buy a flat, the urban-rural divide is not necessarily equated with urban-rural *hukou* divide. Therefore, we focus on whether the house is located in urban areas or rural areas, given that the price appreciation patterns, housing allocation, and transaction are different between the urban and rural housing markets.

## 3. Methods

### 3.1. Data

In order to examine these questions, we draw upon data from the 2016 China Family Panel Studies (CFPS) survey, a nationally representative, longitudinal survey of Chinese communities, families, and individuals conducted by the Institute of Chinese Social Science Survey of Peking University [68]. CFPS implemented its baseline survey in 2010 and conducted three waves of full sample follow-up surveys in 2012, 2014, and 2016. The baseline target sample of CFPS consisted of approximately 16,000 households in 25 provinces, municipalities, and autonomous regions [69], representing 95% of the Chinese population [70]. The 2016 CFPS survey provides the relevant information about homeownership, family assets, and individual psychological well-being. After excluding missing data of the variables concerned in the 2016 adult database, the final sample size for our analyses was 31,368, which includes people between 16 and 98 years of age.

### 3.2. Measures

#### 3.2.1. Outcomes of Interest: Psychological Well-Being

We examine two measures of psychological well-being: depression and life satisfaction. To measure depression, we use the Center for Epidemiologic Studies Depression Scale (CES-D) in the 2016 CFPS questionnaire [71]. Respondents were asked to self-rate their degree of experience to the CES-D 20 questionnaire using a four-point scale: “rarely or never (less than 1 day)”, “not too often (1–2 days)”, “sometimes or half the time (3–4 days)”, and “most of the time (5–7 days)”. The responses for the items of negative feelings (e.g., I find it difficult to do anything) were assigned to an index value of 0, 1, 2, and 3, and those to positive feelings (e.g., I feel happy) were assigned as 3, 2, 1, and 0 [72]. The total score of the respondents ranged from 0 to 51, with higher scores signalling a higher risk for depression.

The measure of life satisfaction was based on the single question: “Are you satisfied with your life?” The response categories followed the Likert scale ranging from (1) very unsatisfied to (5) very satisfied.

#### 3.2.2. Predictor Variables: Homeownership

Our predictor variable, homeownership, was constructed from two questions, “Who owns the house where you and your family currently live?” and “Do you or your family members own any other house than the one where you currently live? (Yes = 1, No = 0)” The former question provided seven categorical responses: (1) solely owned by the family member; (2) partly owned by the family member; (3) public house (*gong fang*) provided by work unit (*danwei*); (4) cheap rental house; (5) public rental house; (6) commercial house rentable in the market; and (7) friends or relatives. We coded homeownership (=1) if the family solely owns the house they currently live in or owns any other house than the one in which they currently live. In our preliminary analysis, we also examined the effect of housing prices on individuals’ psychological well-being. However, the results of our analysis demonstrated that housing prices had no significant impact on psychological well-being between gender or across regions. Therefore, we decided to exclude them from our main models.

#### 3.2.3. Moderators: Gender and Residential Location

In line with the research questions, gender and housing location were used as moderators. Gender was straightforwardly categorized into men and women. Given that urban housing and rural housing are supplied and transacted in different markets, we assume that the psychological benefits of owner-occupied housing as an asset in China depends on where the house owned by the respondent is located. Thus, the housing location was coded as housing located in urban areas (1) and housing located in rural areas (2).

#### 3.2.4. Control Variables

To adjust for potential confounding variables, we include a number of additional features as control variables. Covariates include age, family income (log), level of education, employment status, and household registration status (*hukou*) (Given that holding *hukou* in a particular area is no longer strictly bound with the right to purchase a house in that area, we considered *hukou* as a control variable rather than a moderating variable), marital status, and whether or not an individual is a Chinese Communist Party member (0 no membership, 1 membership). While the individual level of education was measured ordinally, ranging from (1) no formal education/elementary education to (5) post-graduate studies, employment status was constructed as a binary variable (0 no employment, 1 employment). The *hukou* status was coded as 0 for agricultural *hukou* and 1 for urban *hukou*, and marital status is a dichotomous variable that reflects (0) not married and (1) married.

### 3.3. Statistical Analysis

To explore the gender-related differences in the relationship between homeownership and depression, we separately estimated a series of three nested models using ordinary least squares (OLS) regression with robust, clustered standard errors [73] for women (Models 1–5) and men (Models 6–10). For women, Model 1 included no covariates. In Model 2, we adjusted for individual socio-economic and demographic variables. In Model 3, we adjusted for provincial dummies to capture possible geographic heterogeneity. We further conducted subgroup analyses from the full model (Model 3) to examine the association between homeownership and depression depending on residential locations. Model 4 presents results for women in rural areas, while Model 5 presents results for women residing in urban areas. However, the data provide no information about whether the second or subsequent houses that the family owns are located in urban or rural areas. Therefore, for the subgroup analysis regarding residential location (Models 4 and 5), we limited the sample to those who answered the question about the current residence. For men, Models 6 to 10 examine the association between homeownership and depression as well as subgroup analyses in the same order as that for women. Moreover, the gendered differences in the relationship between homeownership and life satisfaction were analysed separately for women and men, using ordered logistic regression with robust standard errors and cluster options. All regression analyses were weighted based on individual-level cross-sectional weights to obtain sample representativeness and more efficient statistical inference [70].

## 4. Results

### 4.1. Descriptive Analysis

Table 1 presents descriptive statistics for all measures. Approximately 50% of the sample (*n* = 15,764) were female, and the average age at baseline was approximately 46. On average, women had a higher depression score (6.97) than men (5.84), and average levels of depression were higher for rural residents than urban residents. Average life satisfaction was slightly higher for women (3.67) than men (3.57), whereas the difference in life satisfaction seemed to be substantively small between rural and urban residents. When including both current residence and other owned homes, approximately 88.9% of the sample respondents were homeowners. Among these homeowners, 54.6% were currently residing in rural areas, whereas about 45.5% owned current residencies in urban areas.

### 4.2. Multivariate Analysis

Table 2 shows the regression estimates from the OLS models that test the impact of homeownership on depression for women and men. For women, the baseline model (Model 1) shows that homeownership is negatively associated with women’s depression, indicating that women who are homeowners are associated with lower depression. The relationship is not significant even after introducing individual sociodemographic controls and provincial dummies. Homeownership also fails to associate with depression either among rural women or urban women significantly. However, it is interesting to note that homeownership has a significant and negative association with men’s depression, particularly in rural areas. According to the baseline model for men (Model 6), men who are homeowners are significantly less likely to show depressive symptoms than non-homeowners. The negative relationship between homeownership and depression among men is consistently significant when introducing individual and provincial-level controls. Moreover, this negative effect of homeownership on the levels of depressive symptoms is more pronounced for men residing in rural areas than in urban areas. As for the personal and household characteristics, younger persons and married people are less likely to have depressive symptoms except among men in urban areas, and those with higher family incomes and education levels tend to have a lower level of depressive symptoms. While urban *hukou* is generally less associated with depression among women, holding a Chinese Communist Party membership has a significantly negative relationship with depression among men.

Table 3 presents the regression estimates from the ordered logistic regression models that examine the relationship between homeownership and life satisfaction. Similar to the results on Table 2, homeownership has a significant effect only on men and not on women. Although the baseline model for women (Model 1) shows that homeownership has a significantly positive influence on women’s life satisfaction, this relationship is no longer significant when including individual control variables and provincial dummies. On the other hand, homeownership has a significantly positive impact on men’s life satisfaction. As can be seen in the full model (Model 8), men who own homes are more likely to be satisfied with their lives than those who are not homeowners. However, the positive impact of homeownership on men’s life satisfaction does not differ between rural or urban areas. In contrast to depression, older persons are more likely satisfied with their lives. Family income is positively associated with life satisfaction, and overall, education and employment have a negative influence on life satisfaction. While the association between urban *hukou* and life satisfaction is inconsistent between genders and regions, married people show a lower level of life satisfaction in urban areas, and Communist Party membership is positively related to life satisfaction.

## 5. Discussion

Using the data from the 2016 China Family Panel Study, we examined how homeownership is associated with depression and life satisfaction in China, and whether this relationship differs between men and women and between urban and rural areas. The results in this study generally support our hypotheses and provide a deeper understanding of the relationship between homeownership and psychological well-being.

This study reaffirmed that homeownership is beneficial to psychological well-being, which has been consistently found in prior studies not only on China (e.g., [49,56,65]), but also on other countries (e.g., [8,27,33]). Given that housing is the largest household asset, our study underpins the social causation theory and asset effect theory, positing that high SES, particularly assets in the form of homeownership, is associated with better mental health [1,16,17,22,74]. In China, housing is no longer state welfare, and purchasing commodified housing has accelerated household wealth accumulation through property value appreciation [75]. Hence, homeownership has increasingly functioned as the material resource that shapes socio-economic status and subjective class identity in China [76]. In this respect, China’s high homeownership rate seems to contribute to psychological well-being.

However, we found that the positive impact of homeownership on psychological well-being is only limited to men, while homeownership has no significant impact on women’s psychological well-being—the psychological benefits of homeownership are more pronounced among men. This result supports that men show steeper socio-economic gradients in health outcomes than women [43]. There could be several explanations for this result. Drawing on Ronald [61] and Macintyre [62] discussed above, the evident relationship between homeownership and psychological well-being among men may be attributable to the fact that the financial security obtained from homeownership (i.e., a ‘hard’ version of the health benefit of material conditions, in Macintyre’s terms) plays a far more significant role in promoting psychological well-being than the psychological security of living in owner-occupied housing (i.e., ‘soft’ version) does.

Another potential reason could be related to the cultural norms of the gender role attitudes in China. As Qing [77] notes, the traditional perception of men as ‘breadwinners’ and women as ‘homemakers’ has contributed to the entrenched expectations of men responsible for household financial resources in China, despite the continued increase in women’s labour participation. Moreover, the likelihood of becoming a homeowner is much higher among men than women [76]. Therefore, the adverse impact of not owning a home might have been so significant that it is linked to psychological well-being for men but not for women.

Our sub-analysis also revealed that the association between homeownership and men’s depressive symptoms is significant only in rural areas, not in urban areas, whereas there is no regional disparity in the linkage between homeownership and men’s life satisfaction. China’s highly dualized institutional structures in land and housing allocation have differentiated the urban and rural housing markets. Despite the increasing urban-rural integration in the past two decades, the slow-going marketization of rural housing amid rapid urbanization in large cities has exacerbated China’s urban-rural housing and wealth inequality. Given that urban housing is usually more expensive than rural housing, our study thus shows a rather surprising result, which also contrasts to Liang’s [66] finding that there is no significant association between homeownership and depression among older people in rural China.

A plausible explanation for this result can be derived from the peculiar rural land allocation system in China. Since state welfare for rural households is largely limited, land-use rights have functioned as the key sources of rural welfare in China [78]. However, only men are entitled to inherit and access rural land rights, and women are excluded from land (re)allocation unless they are widowed. This discriminatory land allocation in rural areas has given men considerable advantages, as well as responsibilities, with regard to obtaining homeownership [79]. Therefore, having no homeownership might mean that male adults in rural areas feel ashamed and less privileged, leading to depressive symptoms. Another explanation can be drawn from social comparison theory, which maintains that people tend to judge their conditions compared to a reference group in the same class or same locality [80,81]. Previous research found that although the property prices in rural areas are lower than those in urban areas, housing wealth inequality in rural areas is more salient than in urban areas, possibly due to the recent increase in rural land values and heterogeneous conditions across different villages [36]. In effect, Li et al. [67] found that peer effects (i.e., strong aspirations for keeping up with their neighbors’ housing size) exist in rural residents’ housing behaviours. Therefore, rural men without homeownership may compare themselves to other rural men with homeownership, and the noticeable housing wealth inequality between them might have had a significantly adverse impact on non-homeowners’ psychological well-being.

## 6. Conclusions

This study advances our understanding of the relationship among assets in the form of homeownership, psychological well-being, and gender. Conceptually, this study contributes to the knowledge of how a biological determinant, i.e., gender, interacts with a social determinant, i.e., homeownership, to affect psychological well-being. Empirically, it draws attention to the potential adverse impact of the constantly increasing housing wealth gap on psychological well-being that has already shown disparity between gender and regions. The paper highlights that in the psychological benefits of homeownership, the materialistic merits of owner-occupied housing could play a significant role, possibly leading to gender disparity in psychological well-being. It also notes that housing wealth inequality could be a more significant factor of psychological well-being than housing wealth per se.

This study has several limitations. First, it used a broad definition of homeownership and measured the household’s homeownership status. It did not differentiate whether or not the respondent is registered as the owner of the house. Considering the gender inequality of homeownership within a household would give additional evidence that validates the materialistic merits of homeownership in promoting psychological well-being. Second, we used cross-sectional data to examine the relationship between homeownership and psychological well-being because the data on depression and life satisfaction was not always collected in the same years in the panel study, which might have resulted in limited causal inferences. Finally, since we used province fixed effects models, the differences between coastal, central, and western provinces or between large cities and small cities were not examined. Using a more refined meaning of homeownership and longitudinal data with considerations of different regional characteristics in future research would advance the knowledge of the relationship of psychological well-being with assets in general and homeownership in particular.

Despite these limitations, the gender and urban-rural differences in the relationship between homeownership, life satisfaction, and depression identified in this study would still provide valuable implications for gender-sensitive policymaking, the land allocation system, and housing market coordination in China. They could also offer insights into policies and practices for promoting psychological well-being in other countries with similar regional and gender disparities in homeownership and land administration systems, such as Southeast Asia and Africa [82,83].

## Figures and Tables

**Table 1:** Summary Statistics of Key Variables (*n* = 31,368).

	Observations	Mean (SD)	%	Min	Max	Gender		Region	
						Women (*n* = 15,764)	Men (*n* = 15,604)	Rural (*n* = 15,947)	Urban (*n* = 15,217)
Depression	31,368	6.41 (5.589)		0	51	6.972 (5.863)	5.843 (5.249)	6.865 (5.840)	5.937 (5.287)
Life Satisfaction	31,368	3.62 (1.077)		1	5	3.668 (1.075)	3.567 (1.076)	3.608 (1.096)	3.630 (1.054)
Homeownership (current + any other residence)	30,171	0.89 (0.314)		0	1				
Yes	26,693		88.9			50.3%	49.7%	53.4%	46.6%
No	3478		11.1			50.1%	40.9%	32.8%	67.2%
Homeownership (current residence)	31,368	0.85 (0.357)							
Yes	26,681		85.1			50.4%	49.7%	54.6%	45.5%
No	4687		14.9			49.7%	50.3%	31.8%	68.2%
Age	31,368	45.80 (16.919)		16	98	45.66 (16.951)	45.95 (16.889)	46.46 (16.817)	45.23 (17.010)
Family Income (log)	31,368	10.78 (1.016)		1.79	16.25	10.78 (1.027)	10.79 (1.006)	10.52 (0.100)	11.05 (0.959)
Education	31,368	1.90 (1.017)		1	5				
None/Elementary	14,474		46.1			55.9%	44.1%	64.4%	35.6%
Junior High	9029		28.8			44.9%	55.1%	48.3%	51.7%
High School	4581		14.6			44.9%	55.1%	36.2%	63.8%
College	3171		10.1			47.9%	52.1%	22.2%	77.8%
Postgraduate	113		0.4			38.9%	61.1%	10.7%	89.3%
Employment	31,368	0.71 (0.455)		0	1				
Yes	22,201		70.8			45.1%	54.9%	55.4%	44.7%
No	9167		29.2			62.7%	37.3%	41.1%	58.9%
Household Registration (registered *hukou*)	31,368	0.26 (0.441)		0	1				
Agricultural *hukou*	23,097		73.6			50.6%	49.4%	64.7%	13.5%
Urban *hukou*	8271		26.4			49.2%	50.8%	35.3%	86.5%
Marital Status	31,368	0.79 (0.410)		0	1				
Married	24,682		78.7			50.5%	49.5%	51.8%	48.2%
Not Married	6686		21.3			49.5%	50.5%	48.7%	51.3%
Party Membership	31,368	0.08 (0.278)		0	1				
Yes	2649		8.4			27.5%	72.5%	37.1%	62.9%
No	28,719		91.6			52.4%	47.6%	52.5%	47.5%

**Table 2:** Homeownership on Depression (cross-sectional weights).

	Women					Men				
	(1)	(2)	(3)	(4)	(5)	(6)	(7)	(8)	(9)	(10)
				Rural	Urban				Rural	Urban
Homeownership	−0.018	−0.002	−0.021			−0.518 *	−0.462 *	−0.491 *		
	(0.237)	(0.226)	(0.230)			(0.229)	(0.223)	(0.225)		
Homeownership (current residence)				0.083	0.072				−0.623 *	−0.311
				(0.276)	(0.261)				(0.315)	(0.238)
Age		0.039 ***	0.043 ***	0.045 ***	0.043 ***		0.019 ***	0.023 ***	0.045 ***	0.010
		(0.005)	(0.005)	(0.006)	(0.007)		(0.005)	(0.005)	(0.007)	(0.007)
Family Income (log)		−0.722 ***	−0.680 ***	−0.662 ***	−0.676 ***		−0.608 ***	−0.552 ***	−0.605 ***	−0.469 ***
		(0.077)	(0.080)	(0.101)	(0.119)		(0.080)	(0.083)	(0.115)	(0.116)
Education		−0.414 ***	−0.373 ***	−0.546 ***	−0.299 **		−0.274 ***	−0.229 ***	−0.457 ***	−0.177 ^+^
		(0.083)	(0.083)	(0.116)	(0.114)		(0.068)	(0.068)	(0.095)	(0.092)
Employment		0.040	0.066	−0.518 *	0.337		0.258	0.257	−0.491 ^+^	0.362
		(0.156)	(0.157)	(0.215)	(0.222)		(0.171)	(0.171)	(0.267)	(0.225)
Household Registration		−0.628 **	−0.562 **	0.195	−0.509 *		−0.311 ^+^	−0.317 ^+^	0.238	−0.361 ^+^
		(0.195)	(0.202)	(0.351)	(0.258)		(0.161)	(0.170)	(0.306)	(0.217)
Marital Status		−0.709 ***	−0.701 ***	−0.562 *	−0.815 **		−0.873 ***	−0.899 ***	−1.529 ***	−0.397
		(0.215)	(0.211)	(0.261)	(0.289)		(0.217)	(0.214)	(0.273)	(0.307)
Party Membership		−0.205	−0.263	−0.464	−0.305		−0.860 ***	−0.936 ***	−0.709 **	−0.954 ***
		(0.303)	(0.305)	(0.426)	(0.373)		(0.187)	(0.188)	(0.238)	(0.244)
Province FE			YES	YES	YES			YES	YES	YES
*R* ^2^	0.000	0.052	0.064	0.072	0.053	0.000	0.042	0.058	0.075	0.041
*n*	14,422	14,422	14,422	7290	7057	14,718	14,718	14,718	7615	6994

Standard errors in parentheses; ^+^ *p* < 0.1, * *p* < 0.05, ** *p* < 0.01, *** *p* < 0.001. (1), (6) Baseline model; (2), (7) Baseline + Individual covariates; (3), (8) Full model: +Individual covariates + province fixed effects; (4), (9) Full model for Rural area; (5), (10) Full model for Urban Area.

**Table 3:** Homeownership on Life Satisfaction.

	Women					Men				
	(1)	(2)	(3)	(4)	(5)	(6)	(7)	(8)	(9)	(10)
				Rural	Urban				Rural	Urban
Homeownership	0.169 *	0.137 ^+^	0.119			0.177 *	0.172 *	0.139 *		
	(0.072)	(0.073)	(0.075)			(0.072)	(0.070)	(0.071)		
Homeownership (current residence)				−0.040	0.133				0.141	0.102
				(0.094)	(0.082)				(0.100)	(0.082)
Age		0.011 ***	0.011 ***	0.013 ***	0.011 ***		0.016 ***	0.016 ***	0.015 ***	0.016 ***
		(0.002)	(0.002)	(0.002)	(0.002)		(0.002)	(0.002)	(0.002)	(0.002)
Family Income (log)		0.149 ***	0.165 ***	0.138 ***	0.198 ***		0.160 ***	0.176 ***	0.131 ***	0.216 ***
		(0.024)	(0.026)	(0.030)	(0.040)		(0.024)	(0.025)	(0.034)	(0.037)
Education		−0.075 **	−0.090 ***	0.020	−0.141 ***		−0.055 *	−0.068 **	−0.001	−0.107 **
		(0.025)	(0.026)	(0.038)	(0.035)		(0.025)	(0.026)	(0.033)	(0.036)
Employment		−0.282 ***	−0.292 ***	−0.346 ***	−0.265 ***		−0.287 ***	−0.298 ***	−0.290 ***	−0.280 ***
		(0.046)	(0.047)	(0.063)	(0.067)		(0.057)	(0.057)	(0.084)	(0.076)
Household Registration		−0.129 *	−0.075	−0.252 *	−0.032		−0.128 *	−0.090	0.047	−0.150 *
		(0.055)	(0.057)	(0.120)	(0.073)		(0.056)	(0.059)	(0.110)	(0.076)
Marital Status		−0.188 **	−0.204 ***	−0.075	−0.252 **		−0.304 ***	−0.315 ***	−0.107	−0.436 ***
		(0.058)	(0.058)	(0.078)	(0.080)		(0.063)	(0.065)	(0.086)	(0.095)
Party Membership		0.256 **	0.246 *	0.359 *	0.224 ^+^		0.287 ***	0.286 ***	0.183 *	0.363 ***
		(0.096)	(0.096)	(0.147)	(0.122)		(0.064)	(0.066)	(0.085)	(0.088)
Province FE			YES	YES	YES			YES	YES	YES
Pseudo *R*^2^	0.0005	0.007	0.012	0.014	0.013	0.001	0.010	0.016	0.018	0.018
*n*	14,422	14,422	14,422	7290	7057	14,718	14,718	14,718	7615	6994

Standard errors in parentheses; ^+^ *p* < 0.1, * *p* < 0.05, ** *p* < 0.01, *** *p* < 0.001. (1), (6) Baseline model; (2), (7) Baseline + Individual covariates; (3), (8) Full model: +Individual covariates + province fixed effects; (4), (9) Full model for Rural area; (5), (10) Full model for Urban Area.

## Data Availability

The data presented in this study are openly available in the China Family Panel Studies website (https://opendata.pku.edu.cn/dataverse/CFPS?language=en).
